# Multiple myeloma in a young female presenting as an aggressive skull-base tumour

**DOI:** 10.4102/sajr.v28i1.2883

**Published:** 2024-07-31

**Authors:** Ursula Lesar, Leon Janse van Rensburg, Siobhan Oelofsen, Kevin McCree, Christelle Ackerman, Razaan Davis

**Affiliations:** 1 Department of Medical Imaging and Clinical Oncology, Faculty of Medicine and Health Sciences, Stellenbosch University, Tygerberg Academic Hospital, Cape Town, South Africa; 2 Department of Radiology and Diagnostics, Faculty of Dentistry, University of the Western Cape, Cape Town, South Africa; 3 Division of Otorhinolaryngology, Faculty of Medicine and Health Sciences, Tygerberg Hospital, Cape Town, South Africa; 4 Division of Anatomical Pathology, Department of Pathology, Faculty of Medicine and Health Sciences, Stellenbosch University and National Health Laboratory Service, Tygerberg Academic Hospital, Cape Town, South Africa

**Keywords:** multiple myeloma, plasmacytoma, young adult, atypical, head and neck, skull-base, epistaxis

## Abstract

Plasma cell neoplasia has a wide presentation of disease (localised or systemic) according to the International Myeloma Working Group. Radiological imaging identifies plasmacytomas as solitary lesions or part of multiple myeloma. We present a rare case of a 21-year-old female who presented with a skull-base tumour.

**Contribution:**

A head and neck plasmacytoma with further lytic bone lesions was confirmed on imaging. This article presents and discusses the clinical, CT, MRI, positron emission tomography (PET)-CT, histology and laboratory findings.

## Introduction

Isolated plasmacytomas are distinct entities to multiple myeloma (MM). Plasmacytomas represent 2% – 5% of plasma cell neoplasia^1^ with two thirds of solitary plasmacytoma being medullary in origin.^2,3^ This neoplasm typically presents during 6th decade of life^4^ and has a male predilection.^5^ Head and neck tumours that extend into the base of skull and adjacent structures result in significant morbidity and mortality because of the impact on vital neural, vascular and aerodigestive structures traversing this space. The differential diagnosis of a skull-base tumour is limited, even more so in a younger patient. The aetiology of head and neck tumours in young adults includes neoplastic and inflammatory causes. It is important for radiologists to consider less common, but treatable pathologies such as plasmacytoma.

## Ethical considerations

Ethical approval to conduct the study was obtained from the University of Stellenbosch Health Sciences Ethics Committee (HEA-2023-26938). Informed written consent was obtained for this case study.

## Patient presentation

A 21-year-old female presented to the emergency centre with uncontrolled epistaxis. She complained of a 3-month history of recurrent epistaxis. The haemoglobin level was 10 g/L on presentation. The patient had no significant past medical history. Clinically, she had left-sided proptosis with no cranial nerve neuropathy. Endoscopy revealed a pulsatile, vascular mass filling the left nasal passage, extending through the septum into the right nasal cavity. The mass did not involve the posterior nasal space.

### Imaging findings

**FIGURE 1:**
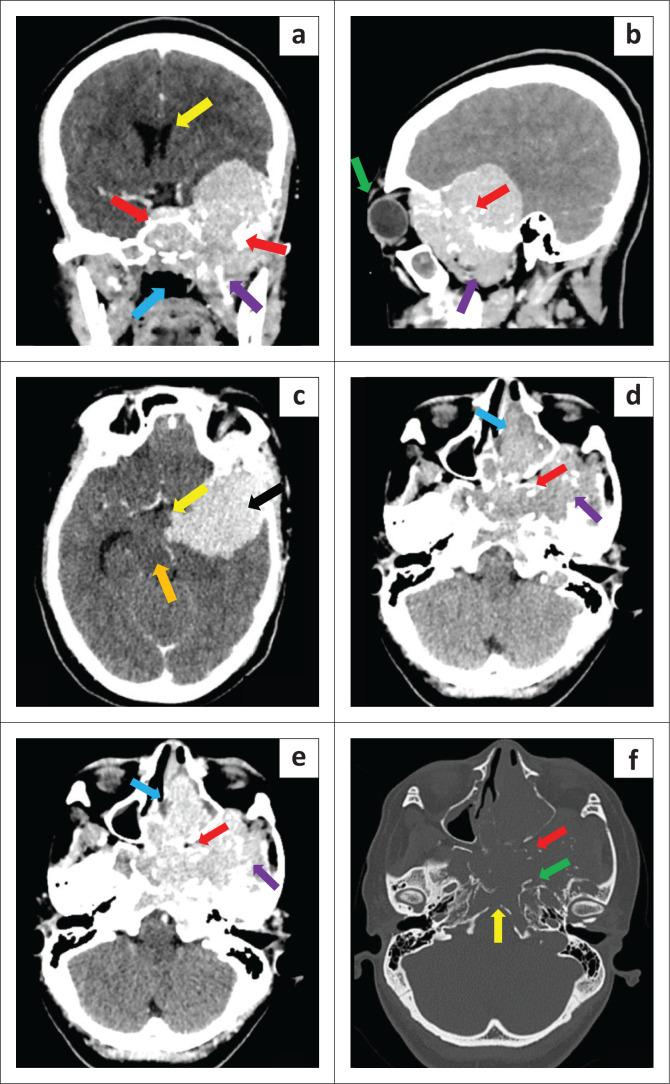
(a, b) Post-contrast coronal image shows intracranial extension of the skull base tumour causing mass effect and compression of the left lateral ventricle (yellow arrow). It invades the central skull base and sphenoid sinus (red arrow), extending inferiorly into the masticator space (purple arrow) and nasopharynx (blue arrow). There is left-sided proptosis (green arrow). (c) Post-contrast axial image shows the extent of the intracranial component (black arrow) of the tumour compressing the midbrain (orange arrow) and extending into the suprasellar cistern (yellow arrow). No cerebral oedema. (d–f) Pre-contrast, post- contrast and bone window images in the same axial plane. The tumour is pre-contrast dense (d) and avidly enhances (e). It involves the left sinonasal passage (blue arrow), left masticator space (purple arrow) and the left pterygopalatine fossa (red arrow). Erosion of the bone (f) including the pterygoid plates (red arrow), clivus (yellow arrow) and petrous apex (green arrow).

A CT study of the head showed a pre-contrast dense, post-contrast avidly enhancing, locally destructive mass centred in the left skull base with intracranial and left intraorbital extension, consistent with a tumour. The tumour eroded the left temporal, sphenoid, clivus and pterygoid bones. The radiodense material within the matrix was in keeping with scattered bone fragments (Figure 1).

**FIGURE 2:**
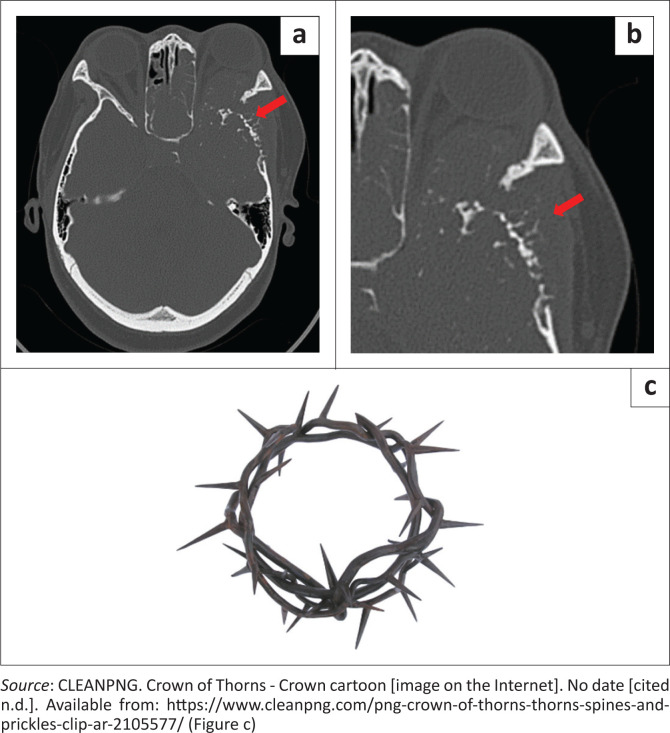
Bone window (a) and magnified bone window (b) of the left temporal bone show the expansion of the medullary cavity and unusual periosteal reaction (red arrow) that is perpendicular to the distorted cortex and has an irregular curled and haphazard construction reminiscent of a crown of thorns (c).

There was expansion of the medullary compartment of the flat bones of the skull with an unusual periosteal reaction perpendicular to the distorted cortical margin that was haphazardly curled and irregular (Figure 2^6^).

**FIGURE 3:**
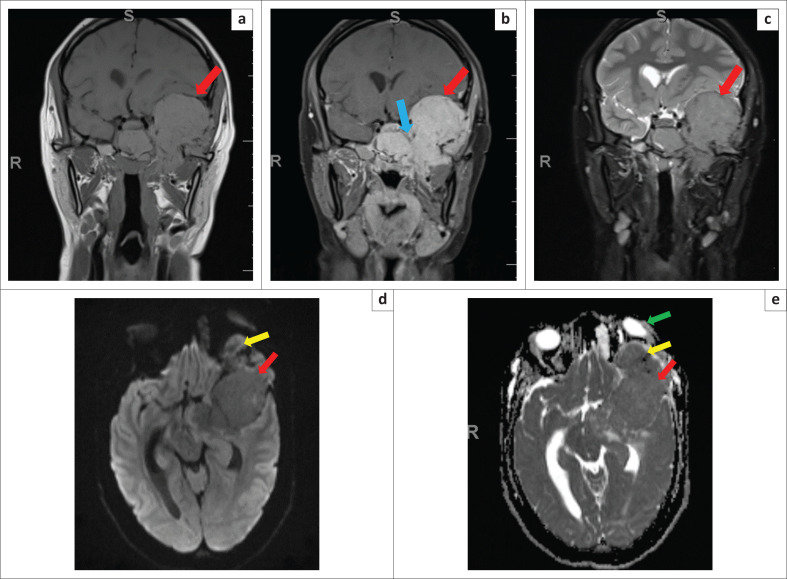
(a–c) Coronal pre-contrast T1-weighted (T1W), post contrast T1W fat saturation (FS) and T2-weighted (T2W) FS at the same level. The trans-spatial base of skull tumour (red arrow) is hyperintense to muscle on T1W (a) and T2W FS (c) and avidly enhances on post contrast T1W FS (b). Invasion of the left cavernous sinus is demonstrated (blue arrow). (d–e) No restricted diffusion (red arrow) on axial diffusion-weighted imaging (DWI) (d) and apparent diffusion coefficient (ADC) (e). The tumour is shown to extend into the extraconal compartment of the left orbit (yellow arrow) with resultant proptosis of the left globe (green arrow).

MRI of the brain confirmed a trans-spatial tumour that was hyperintense on T1-weighted (T1W), isointense on T2-weighted (T2W) and fluid-attenuated inversion recovery (FLAIR) images when compared to muscle and avidly enhancing on T1W-post-gadolinium images. The tumour showed no restricted diffusion on apparent diffusion coefficient (ADC)/diffusion-weighted imaging (DWI) (Figure 3). The aggressive nature of the tumour was further highlighted by the intracranial extent with cavernous sinus infiltration (Figure 3).

**FIGURE 4:**
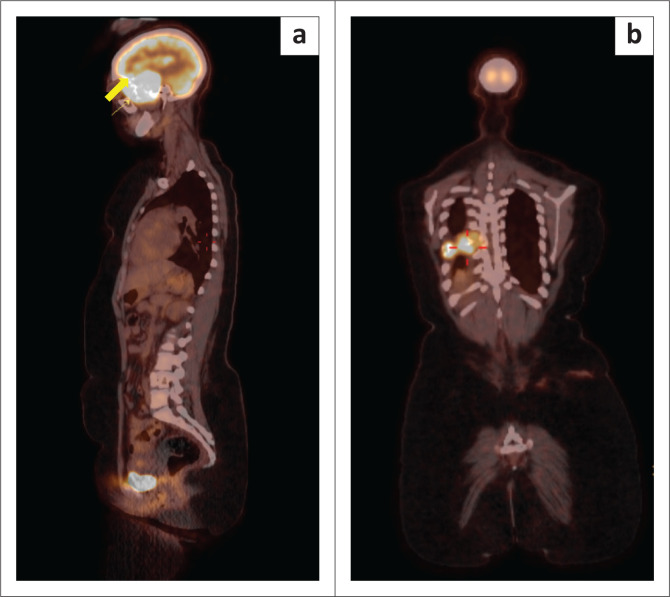
(a, b) ^18^F-FDG positron emission tomography (PET)/CT demonstrates the avid left skull-base tumour (yellow arrow) (a) and the avid focus in the right posterior 8th rib (red cross hairs) (b).

The tumour was biopsied, and histology suggested a plasmacytoma. As per the International Myeloma Working Group (IMWG) protocol, a ^18^F-fluorodeoxyglucose (^18^FDG) positron emission tomography (PET)/CT was performed to assess for other associated lesions. ^18^FDG PET-CT showed intense uptake in the left base of skull mass with intracranial extension. There was an associated lytic, expansile lesion in the right posterior 8th rib and infiltration of the adjacent 8th thoracic vertebra. Furthermore, avid uptake in a lytic lesion in the 11th thoracic vertebral body was demonstrated (Figure 4).

### Laboratory findings

Blood tests indicated a normal serum urea, creatinine and calcium. The HIV antigen test was negative. Light microscopy of the mass revealed a lymphoproliferative neoplasm characterised by sheets of plasma cells, with a notable proportion that displayed a less mature phenotype. Many cells exhibited an eccentrically located nucleus, abundant amphophilic cytoplasm, and a distinctive paranuclear hof, which gave rise to a clock face appearance. Nucleoli were frequently observed, ranging from one to multiple per cell. Mitotic figures were occasionally identified. The background fibrous tissue exhibited fibrosis and haemosiderin deposition was evident. No calcification was observed in this tissue sample.

**FIGURE 5:**
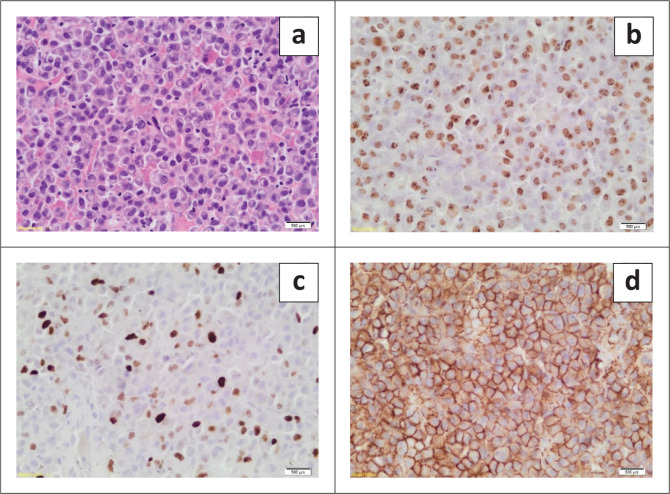
(a–d) Many cells exhibited an eccentrically located nucleus, abundant amphophilic cytoplasm, and a distinctive paranuclear hof, which gave rise to a clock face appearance (a). The lymphoproliferative neoplasm displayed negative staining for CD20, CD3, and CD56 (not shown). Epstein–Barr encoding region (EBER) in situ hybridisation yielded a positive result (b). The Ki67% was interpreted to be 20% – 30% (c), and plasma cell markers were positive, including CD138 (d), multiple myeloma oncogene-1 (MUM1), and VS38c. Kappa and Lambda in situ hybridisation revealed a Lambda light chain restriction, consistent with a neoplastic proliferation of plasma cells (not shown).

A solitary plasmacytoma of the bone was favoured, but plasma cell myeloma needed to be excluded (Figure 5). Serum protein electrophoresis showed a peak in the gamma region, quantitated as 43 g/L. Serum immunofixation demonstrated an immunoglobulin G (IgG) lambda monoclonal gammopathy. Bone marrow aspirate and trephine exhibited no increase in plasma cells.

### Management and outcome

**FIGURE 6:**
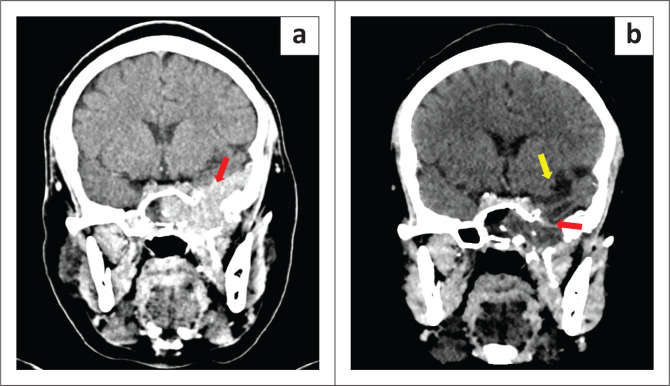
(a, b) Post-therapeutic post-contrast coronal CT head at 5 months (a) and 9 months (b) after diagnosis. Post-chemotherapy image (a) shows a decrease in the volume of the base of skull plasmacytoma (red arrow). Post-radiation image (b) shows a radiological response with no residual enhancing tumour (red arrow). There is post-therapeutic left temporal lobe volume loss (yellow arrow).

In view of the multiple rib and thoracic vertebral lesions confirmed on F-18 FDG PET-CT, the patient was diagnosed with MM and completed three cycles of chemotherapy (cyclophosphamide, thalidomide, and dexamethasone). A decrease in volume of the base of skull plasmacytoma on follow up CT imaging was in keeping with a partial response to therapy (Figure 6a). The patient received a further 15 fractions of radiotherapy to the base of skull mass which resulted in a complete radiological response with no appreciable mass on follow up imaging (Figure 6b). Clinically, the patient’s proptosis improved and there was no further epistaxis. The patient remains under the care of the Department of Haematology for further monitoring.

## Discussion

In 2014, the IMWG revised the diagnostic criteria for MM and related disorders. Web based MM calculators, using the IMWG criteria, serve as tools to assist its application.

Plasmacytomas can occur in isolation or in conjunction with MM and radiologically include solitary bone plasmacytoma (SBP), extramedullary (soft tissue) plasmacytoma (EMP) and diffuse disease, MM.^7^ In contrast to MM, isolated plasmacytomas have no further bone marrow involvement. Plasmacytoma and MM are an immunoproliferative monoclonal disease of the B-cell line, which originate from neoplastic transformation of plasma cells. Typical presentation of MM is in the 5th to 8th decade of life with a male predominance.^4,5^

Plasmacytoma of the skull base is rare with few cases described in the literature.^8^ The presenting symptoms depend on site of disease.^8^ The diagnosis of MM is based on tissue biopsy, immunohistology staining and full body imaging. In this case, a biopsy proven solitary bone plasmacytoma of the left base of skull and the multiple osteolytic lesions identified on PET-CT fulfilled the diagnostic criteria of MM.^7^

At the time of presentation and CT imaging, the differential diagnosis included lymphoma, aggressive meningioma, nasopharyngeal carcinoma, primary osteosarcoma and chondrosarcoma. Lymphoma was excluded because of absent MRI restricted diffusion. Although this avidly enhancing tumour extended along the dural margin with a ‘mushrooming’ appearance^9^ it lacked the adjacent bone hyperostosis expected with meningioma. Nasopharyngeal carcinoma is rare in a patient under the age of 30 years^10^ and would be associated with lymphadenopathy, which was absent in this case. Although primary osteosarcoma of the skull bones is a rare entity^11^ the soft tissue tumour with an atypical sunburst periosteal reaction supported the inclusion of osteosarcoma in the differential diagnosis. Chondrosarcoma was considered in view of the scattered irregular radiodense material that was interpreted as a chondroid matrix, which on review was consistent with bone fragments. Chondrosarcoma is the second most common bone tumour and usually presents in patients during the 3rd – 6th decade of life.^12^ Although rare in the adolescent and young adult (15 years – 39 years), chondrosarcoma has been documented in approximately 16% of bone sarcomas in this age group.^12^ A further characteristic finding of note is the unusual periosteal reaction along the flat bones of the skull. The periosteal reaction that is perpendicular to the distorted cortical margin with a haphazard curled and irregular appearance has been reported in the literature as an unusual sunburst or sun ray found in the sternum, ribs and mandible.^13,14,15^ This periosteal reaction is distinctive and reminiscent of a crown of thorns appearance (Figure 2a–2c)^6^. The authors therefore suggest that plasmacytoma is considered when this crown of thorns periosteal reaction is encountered.

Skull-base plasmacytomas have a mean age of presentation in the 6th decade^4,13^ and 5% of patients with MM have an initial diagnosis of solitary plasmacytoma.^16^ Only 0.3% of all patients diagnosed with MM are diagnosed before 30 years of age.^17^ The common locations of base of skull plasmacytomas in descending order are sphenoclival, nasopharynx, petrous roof and orbital roof.^8^ In the sphenoclival region, the signs and symptoms at presentation are diplopia, hemianopia, blurred vision, ocular pain and cranial nerve neuropathy of cranial nerves II, III, IV, VI, IX and X.^8,18,19^ Recurrent epistaxis is typically observed in nasopharyngeal plasmacytomas.^8^ In this case, the mass at presentation had extensive involvement of the base of skull that included the nasal cavity and orbit.

Multiple myeloma employs conventional radiographs as a reference standard for diagnosis as these are cheap and easily available. Diagnosis of osseous and extraosseous manifestations of MM requires cross-sectional imaging modalities such as whole-body low-dose CT, whole-body MRI and ^18^F-fluorodeoxyglucose (FDG) PET-CT.^20^ MRI is the modality of choice to detect bone marrow involvement, while PET-CT has prognostic data and aids in response to therapy.^20,21^ Conventional radiography is being replaced by whole-body low-dose CT in some institutes because of its higher sensitivity for the detection of osseous lesions and its ability to diagnose extraosseous lesions.^20^ The modalities with the highest sensitivity for both detection of bone marrow disease and extraosseous lesions are whole-body MRI and ^18^F-FDG PET-CT.^20,21^ In this study, CT and MRI head was initially used. Once the tissue biopsy confirmed a skull-base plasmacytoma, according to our institutional clinical protocol, ^18^F-FDG PET-CT was used to assist in assessing for MM and the presence or absence of lytic lesions elsewhere in the skeleton. MRI is a more sensitive method for initial staging of MM, while ^18^F-FDG PET-CT allows monitoring of therapy of MM.^20^

## Conclusion

This case highlights the diagnosis of MM in a young patient under the age of 30 years presenting with an aggressive base of skull mass and epistaxis. It underscores the atypical periosteal reaction seen in flat bones reminiscent of a crown of thorns that we suggest should prompt the inclusion of plasmacytoma in the differential of a skull-base tumour. Furthermore, this case emphasises the role of mulitmodality whole-body imaging in assessing MM.
